# Personal

**Published:** 1900-03

**Authors:** 


### PERSONAL.

DR. JOHN W. CAMERON, of Buffalo, has in connection with his general practice, opened an office at 46 W. Huron Street, for the treatment of diseases of the nose and throat. Hours: 12 to 3 p. m. His office for general practice is at his residence, 159 Woodlawn Avenue. Hours: 8 to 9 a. m. and 7 to 8 p. m.

---

DR. GEORGE S. PALMER, of 325 Franklin Street, Buffalo, who had but recently recovered from an illness in December, is reported ill with pneumonia. We trust he is in a fair way to recover with reasonable promptitude.

